# p120RasGAP Is a Mediator of Rho Pathway Activation and Tumorigenicity in the DLD1 Colorectal Cancer Cell Line

**DOI:** 10.1371/journal.pone.0086103

**Published:** 2014-01-21

**Authors:** Shawna L. Organ, Josephine Hai, Nikolina Radulovich, Christopher B. Marshall, Lisa Leung, Takehiko Sasazuki, Senji Shirasawa, Chang-Qi Zhu, Roya Navab, Mitsuhiko Ikura, Ming-Sound Tsao

**Affiliations:** 1 Princess Margaret Cancer Centre, Toronto, Ontario, Canada; 2 Department of Laboratory Medicine and Pathobiology, University of Toronto, Toronto, Ontario, Canada; 3 Department of Medical Biophysics, University of Toronto, Toronto, Ontario, Canada; 4 Department of Pathology, Research Institute, International Medical Center of Japan, Tokyo, Japan; 5 Department of Cell Biology, School of Medicine, Fukuoka University, Fukuoka, Japan; University of San Francisco, United States of America

## Abstract

*KRAS* is mutated in ∼40% of colorectal cancer (CRC), and there are limited effective treatments for advanced *KRAS* mutant CRC. Therefore, it is crucial that downstream mediators of oncogenic KRAS continue to be studied. We identified p190RhoGAP as being phosphorylated in the DLD1 CRC cell line, which expresses a heterozygous *KRAS* G13D allele, and not in DKO4 in which the mutant allele has been deleted by somatic recombination. We found that a ubiquitous binding partner of p190RhoGAP, p120RasGAP (RasGAP), is expressed in much lower levels in DKO4 cells compared to DLD1, and this expression is regulated by KRAS. Rescue of RasGAP expression in DKO4 rescued Rho pathway activation and partially rescued tumorigenicity in DKO4 cells, indicating that the combination of mutant KRAS and RasGAP expression is crucial to these phenotypes. We conclude that RasGAP is an important effector of mutant KRAS in CRC.

## Introduction

In North America, colorectal cancer (CRC) is the third most prevalent form of cancer in both men and women. In 2013, it is estimated that over 100,000 new cases will be diagnosed in the United States, resulting in over 50,000 deaths [1]. Although the rate of death from colorectal cancer has declined by 3% over the past ten years [1], metastatic disease, most prominently to the liver, will develop in 30% to 40% of CRC patients, and 50% will die of CRC recurrence [2]. Surgical resection is the standard for treatment of early stage CRC, but limited effective therapies are available for advanced patients [3]. The development of CRC involves a multistep process with the accumulation of both genetic and epigenetic changes, including alterations of the KRAS pathway [4]. *KRAS* activating mutations occur in approximately 40–50% of CRC, with the most common mutations being found in codon 12 (∼80%) and codon 13 (∼20%).

Currently, the newest approved treatments for CRC are with the targeted epidermal growth factor receptor (EGFR) inhibitors, such as cetuximab and panitumumab, in combination with chemotherapy. However, only patients with wild-type *KRAS* derive significant clinical benefit from this treatment, as those with *KRAS* mutations do not show a significant survival benefit [5]. Therefore, current studies are aimed at finding novel downstream effectors of mutant *KRAS* that can be used in combination to inhibit signaling from this pathway.

The activity of wild-type RAS is closely controlled by families of GTP-ase activating proteins (GAPs), which inactivate RAS by facilitating the hydrolysis of bound GTP, and GTP exchange factors (GEFs), which facilitate the release of GDP so that RAS can once again bind GTP[6]. Of the large family of RasGAPs that are now known, one of the earliest identified and most extensively studied is p120RasGAP, or simply RasGAP, the product of the *RASA1* gene [7], [8]. Disruption of the *RASA1* gene in mice results in embryonic lethality at E10.5, due to aberrant cardiovascular system development [9]. Transgenic mouse embryos created from RNAi-mediated *RASA1* knockdown in ES cells demonstrated that the severity of vascular defects correlated with the level of residual RasGAP expression, and mosaic embryos develop localized defects [10]. Consistent with these mouse studies, mutations in the *RASA1* gene have been linked with familial capillary venous malformation syndromes which can present with a wide range of phenotypes, most commonly that known as a “port wine stain” [11], [12], [13], [14], [15]. Recent proteomic analysis of these skin lesions showed consistent decreased expression of RasGAP compared to surrounding normal tissue [16]. This together suggests that *RASA1* plays a crucial role in angiogenesis and vascular development. However, although protein modulation of RasGAP has been found in several neoplasms including chronic myelogenous leukemia [17], astrocytoma [18], trophoblastic tumors [19], prostate cancer [20], liver cancer [21], and basal cell carcinoma [22], protein levels have not necessarily been found to be correlated with RAS activity or cancer severity [22], [23]. Therefore, the role of RasGAP in cancer remains to be clarified.

The SH2-SH3-SH2 domain configuration in the N-terminal region of RasGAP has long suggested to researchers that RasGAP could play a role as a signaling adaptor protein, by contributing to, as well as being independent of, its GAP activity [7], [24]. Importantly, these domains were found to bind to tyrosine phosphorylated p190RhoGAP (here referred to as RhoGAP) in response to upstream kinase activity and cell adhesion [25], [26], [27]. This finding provided the first mechanistic evidence for a link between RAS activation and Rho pathway signaling. Our group has recently found that RhoGAP becomes tyrosine phosphorylated downstream of c-MET signaling in the DLD1 mutant *KRAS* CRC cell line [28]. We therefore sought to determine the role of active KRAS in the RhoGAP-RasGAP interaction, and the effect of this interaction in CRC tumor cells.

## Experimental Procedures

### Cell culture

DLD1 (ATCC, Manassas, VA) is a colorectal cancer cell line that is heterozygous for the G13D *KRAS* mutation. DKO4 cells were derived from DLD1 by disruption of the mutant *KRAS* allele by somatic recombination [29]. Both cell lines were routinely grown in Dulbecco's Modified Eagle's Media (DMEM) supplemented with 10% fetal bovine serum (FBS).

### Modulation of gene expression

The RASA1 overexpressing lentiviral vector was constructed using the Gateway Recombination System (Invitrogen, Life Technologies, Carlsbad, CA). Entry vector containing either CMV promoter or RASA1 ORF (OpenBiosystems, ThermoScientific, Ottawa, ON) were recombined with pLenti-CMV-GFP-DEST vector (Addgene plasmid 19732) creating pLentiCMV and pLentiRASA1. HEK293T cells were transfected with these pLenti vectors and lentiviral packaging vectors as described previously [30]. Viral supernatants were collected, filtered, and used to infect target cells in the presence of 4 µg/mL polybrene (Sigma-Aldrich, St. Louis, MO). 72 hours after transduction, cells were sorted for GFP expression. For KRAS mutant overexpression, retroviruses were generated by transfecting Phoenix ecotropic packaging cells with the retroviral vector pBabepuro containing either wild-type KRAS-4B, the mutant *KRAS* constructs, or empty vector using FuGENE 6 transfection reagent (Promega, Madison, WI). Retroviral supernatants were collected as above, and cells were selected with 0.5 µg/mL puromycin (ICN Biomedicals, Irvine, CA) until no untransfected control cells were left alive.

### Immunoprecipitation and Western Blotting

Cells were lysed in a 1% Triton-X 100 buffer (1% Triton X-100, 10% glycerol, 50 mM Hepes, 150 mM NaCl, 1.5 mM MgCl2, 10 mM sodium pyrophosphate, 100 mM NaF, 10 mM Na_4_P_2_O_4_, 1 mM EDTA, 1 mM sodium orthovanadate plus a cOmplete mini EDTA-free protease inhibitor tablet (Roche, Laval, QC), allowed to rest on ice for 10 minutes and then cleared by centrifugation at 14,000 g at 4°C for 30 mins. Protein concentration standardization was performed using Bradford protein assay reagent (Bio-Rad, Hercules, CA). For immunoprecipitations, protein concentration was equalized among samples, and lysates were combined with 2–5 µl of antibody and allowed to rock overnight at 4°C. 30 µl of Protein G PLUS-agarose beads (Santa Cruz Biotechnology, Santa Cruz, CA) were combined with the lysates and rocked for 1 h at 4°C. Immunoprecipitated proteins were then washed 3x with lysis buffer, eluted with 20 µL of 2xSDS sample buffer and boiled for 5 minutes. Whole cell lyates were normalized for protein concentration, combined with 6xSDS sample buffer and boiled for 5 minutes as well. Lysates were then loaded onto a 4–20% gradient SDS polyacrylamide gel (Bio-Rad) and run at 120 V. Gels were transferred onto PVDF membranes before being blocked with either 5% dry skim milk in TBST or 5% BSA in TBST for 1 hour at room temperature and then probed overnight with appropriate antibody. Westerns were probed with appropriate secondary antibody, reacted with ECL prime (GE Healthcare, Piscataway, NJ) and exposed to XRAY film for the appropriate amount of time. Antibodies were used as directed: RasGAP clone B4F8 and anti-phosphotyrosine clone 4G10 (EMD Millipore, Billerica, MA), p190A-RhoGAP (Cell Signaling, Danvers, MA), GAPDH and KRAS 4B clone F234 (Santa Cruz Biotechnology).

### Quantitative Real-Time PCR

Total RNA (1–2 µg) was isolated from cell lines and tissues using a Qiagen RNeasy kit (Qiagen, Hilden, Germany) and was reverse-transcribed using with SuperScript III reverse transcriptase (Invitrogen, Life Technologies, Burlington, ON). A 10 ng equivalent of cDNA was used for each quantitative PCR (qPCR) assay performed with the Stratagene Mx3000p Sequence Detection System using SYBR green 2× master mix. Primers used are:

GAPDH F – CCCCCACCACACTG,

GAPDH R – GCCCCTCCCCTCTTCAAG

RPS13 F - GTTCTGTTCGAAAGCATTG

RPS13 R – AATATCGAGCCAAACGGTGAA

RASA1 F – GGACGAAGGTGACTCTCTGGAT

RASA1 R – GGAGGAGCGGTCAACGGTAT

KRASF – CAGGCTCAGGACTTAGCAAGAAG

KRASR-TGTTTTCGAATTTCTCGAACTAATGTA

Predicted PCR product sequences were verified by using BLAST for recognition of target and non-target sequences. Results were analyzed using the delta-delta Ct method, normalizing against the average of two housekeeping genes.

### Cell-based assays

#### Cell counting

5×10^3^ cells were plated in full serum media, in triplicate, for 5 days of counting in a 24 well plate. Beginning at 72 hours after plating, 3 wells of each cell line are trypsinized and then counted using a Beckman Coulter Z2 cell counter (Beckman-Coulter, Brea, CA).

#### Cell adhesion assay

1×10^5^ cells were seeded onto a 24-well dish coated with 0.01% PureCol collagen (Sigma-Aldrich) for 15 minutes. The wells were stained with 0.2% crystal violet and lysed with 0.1% Triton X-100. The lysate was read at 590 nm on a Tecan XFlour4 plate reader (Mannedorf, Switzerland).

#### Cell motility assay

7×10^4^ cells were plated in 70 µL of full serum media into each side of an µ-dish cell culture insert (Ibidi, Planegg, Germany) in a 24-well cell culture plate and allowed to grow for 72 hours or until a confluent monolayer was reached. The insert was removed and media was changed for serum-free DMEM. Phase contrast images were acquired on the Zeiss Axio Observer was used to take a photograph at 32× magnification at this point (time 0) and every 24 hours thereafter. Image analysis was performed as described previously [31]. Image-Pro Plus software (MediaCybernetics, Rockville, MD) was used to analyze the wound-healing assays. Using edge and segmentation filters, areas with large pixel intensity variations (cells) appear light, whereas smooth areas of the image (wound) appear dark. The filtered image was then converted to a binary image by applying a pixel threshold and the wound area was determined by counting the sum of pixels assigned. The pixel sum was then expressed as percent wound closure, where zero pixels in the wound represents 100% closure.

### Immunofluorescence

Glass chamber slides (BD Biosciences, San Jose, CA) were coated overnight at 4°C with 0.01% collagen in PBS. Trypsanised cells were resuspended in DMEM + 5% BSA, plated on slides and allowed to adhere overnight. Slides were then washed once with PBS and fixed with paraformaldehyde for 20 mins at room temperature. Cells were permeabilized with 0.01% Tween-20 in PBS for 20 mins, blocked with 3% BSA in PBS and stained with 1∶300 rhodamine phalloidin for 1 h at room temperature. Glass coverslips were applied with Vectashield containing DAPI (Vector Laboratories, Burlingame, CA) for nuclear staining. Images presented here were taken at 43× magnification with an oil immersion lens on a Zeiss LSM 700 confocal microscope.

### CRC sample collection

Patient tissue samples were obtained from the UHN snap-frozen tissue bank following approval by the UHN Research Ethics Board. All tissues were collected within 30 min of resection and snap-frozen in liquid nitrogen as well as being formalin fixed and paraffin embedded (FFPE), and their quality has been verified by histology. Tissues included 63 primary colorectal cancers and 30 metastatic colorectal cancers. Metastatic tumors were from liver (22 cases) and lung (8 cases) metastasectomy specimens.

### RASA1 mutation analysis

cDNA was isolated from patient tissues cell lines as described above from total RNA. *RASA1* cDNA was first amplified with 6 sets of primers to cover the length of the gene, and sequencing was performed on the amplified DNA with these same corresponding primers, which are as follows:

F1- CTCAGCCTGGGGAGCTGAAGG

R1 - TGGAGGAGCGGTCAACGGTATG (bp 2–649)

F2 - GGCCTCGGGACAGTGGACGA

R2 - GGGCCTCACAAGAAAACTGCAGAC (bp 563–1252)

F3-AGGTGGGCCGGGAAGAAGATCC

R3-TCCAATCCTCTGCTTGTTCTGGAGT (bp 1131–1823)

F4-TGGCAGGCCAAACTGTTTTCAGA

R4–TGCTGGCCAGTAGTGTTCGGT (bp 1723–2381)

F5-CCGAACACTACTGGCCAGCATCC

R5 - TGACACCTTCCATGTAGGGCTCC (bp 2362–2987)

F6-CGACTCATCTGTCCTGCCATCCT

R6 - CTGGGGCGAAGGCTGCTACC (bp 2825–3277)

For PCR amplification, 0.3 ul each of the forward and reverse primers (50 uM) were added to 6 ul of cDNA (20 ng/ul), 12.5 ul of 2x Taq select DNA polymerase, 0.2 ul of 25 mM dNTP and ultra-pure water (Sigma-Aldrich) to a total volume 25 ul for each reaction. The cycling conditions were: 95°C for 10 min, followed by 39 cycles, with denaturing at 95°C for 45″, annealing at 64°C for 45″, and extension at 72°C for 1 min, a 10 minute incubation at 72°C followed by a 4°C. 5 ul of PCR product was checked on a 2% agarose gel. The PCR product was cleaned up with ExoSAP-IT (Affymatrix, Santa Clara, CA).

To validate sequencing in genomic DNA, the same methods were used as above, using the following primers to amplify and sequence exon 16:

F – CGCTGCCAGTTGAGCCGATTACA

R - CTCTGGCATCATTGTGCTACTAAGC

### Real-Time NMR GAP activity assay

Labeled GTPase domain of RAS 1-171 were expressed from pET15b vectors in *E. coli* in M9 media supplemented with ^15^N ammonium chloride and purified by Ni-NTA affinity chromatography. His tags were removed by thrombin cleavage and monomeric GTPases were further purified by gel filtration chromatography (Superdex 75) [32], [33]. Samples were concentrated, and if necessary exchanged into NMR buffer (e.g., 25 mM HEPES pH 7.0, 100 mM NaCl, 5 mM MgCl_2_, 1 mM DTT, and 10% D_2_O).

To assay intrinsic GTP hydrolysis, a GTP-loaded sample was prepared by incubating the RAS protein (∼10 min at 37°C or longer at room temperature) in the presence of 10-fold molar excess GTP and 10 mM EDTA [34]. Following the exchange, MgCl_2_ is added to a final concentration of 20 mM to stabilize the newly bound nucleotide, and the sample passed through a gel filtration or desalting column (PD MidiTrap™ G-25 (GE Healthcare) equilibrated with NMR buffer to remove excess nucleotide and the eluted sample was then quickly concentrated and snap frozen.

Cells were harvested by scraping in a minimal volume (150 µl for a 10 cm plate) of lysis buffer as described for Western blotting, then cleared by brief centrifugation (16,000 g for 30 s) and the total cellular protein in the supernatant was analyzed using the Bradford assay reagent (BioRad) to standardize the amount of protein used in each assay. A concentration of 10–20 µg/µl total protein in the lysate was achieved and 35 ug in 3.5 µl was added to the purified GTP-bound RAS fragment.

Data collection was subsequently initiated as rapidly as possible. Half lives of reactions were initially estimated by visual inspection of spectra, then, the fraction of GDP-bound GTPase present at each time point was assessed from several pairs of peaks and the data was fitted to a single-phase exponential decay function to obtain the exchange/hydrolysis rates [35].

### RAS activity assay

Cells were serum starved for 24 hours and then assayed for active RAS using the RAS activation kit (Millipore) as directed. Briefly, cells were lysed in 1x MLB lysis buffer and equal protein amounts were mixed with the RAS-binding domain of RAF1 fused to glutathione-*S*-transferase and coupled to glutathione-sepharose beads. After rocking at 4°C for 1 h, the beads were washed in the same lysis buffer and resuspended in 2x SDS sample buffer. Western Blotting proceeded as described above.

### Subcutaneous tumorigenicity assay

#### Ethics Statement

All manipulations were done to minimize animal suffering, in accordance with protocols approved by the Ontario Cancer Institute (OCI) Animal Care Committee under the animal use protocol number AUP 736.9.

Severe combine immunodeficient (SCID) mice were bred on site and obtained from the Ontario Cancer Institute (OCI, Toronto, ON). One million cells were injected subcutaneously in the right shoulder region of 4- to 6-week-old male SCID mice (n = 5–8 per cell line). Once tumors were palpable they were measured every 3 days until humane endpoint was reached, which was either when the tumors reached 1.5 cm, or when they became ulcerated to the point of animal distress. Tumor volume was measured using the formula (length x width^2^) x π/6. Mice were euthanized using CO_2_, as approved by the OCI Animal Care Committee, tumors were excised, and portions were either snap frozen in liquid nitrogen for DNA and protein isolation or fixed in formalin for paraffin embedding and immunohistochemical staining. For statistical analysis, a linear mixed effects (LME) model was used to incorporate the high correlation occurring among measurements taken on the same mouse. All of the tumor volume measurements were square-root transformed to stabilize the variance, and Wald p-value was used to indicate significance.

## Results

### Loss of RasGAP leads to loss of RhoGAP phosphorylation

**Figure 1:**
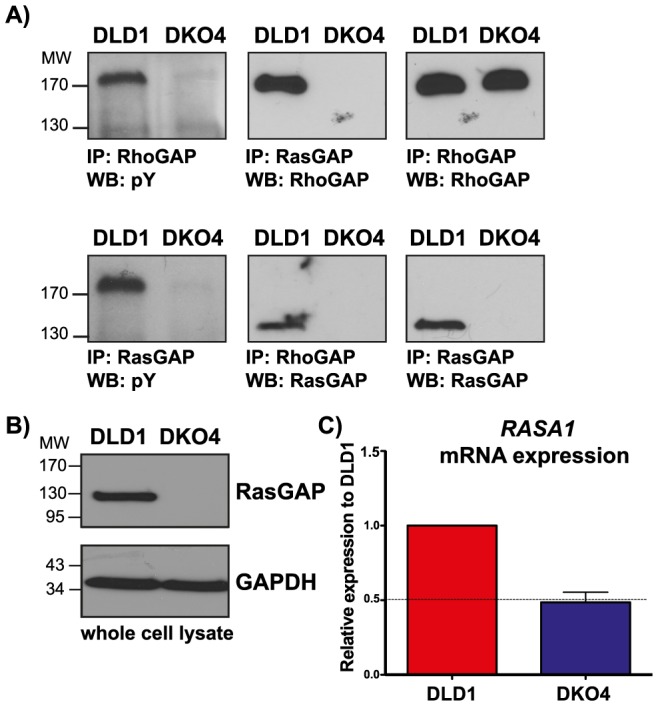
RhoGAP phosphorylation and RasGAP expression in DLD1 and DKO4 cell lines. A) RhoGAP and RasGAP were immunoprecipitated from DLD1 and DKO4 cells. Immunoprecipitates were subjected to Western blotting and probed for total phosphotyrosine, RasGAP and RhoGAP. Western Blotting of whole cell lysates (A) and rt-QPCR (C) were used to determine total protein and mRNA levels respectively in these cell lines.

To further study the role of RhoGAP phosphorylation in DLD1 cells, we investigated its interaction with RasGAP, one of its major binding partners. At the same time, we wanted to know if the loss of mutant *KRAS* in the DKO4 isogenic derivative cell line would have an effect on this interaction. We found that RhoGAP could only be phosphorylated in DLD1 cells, not in DKO4, while total levels of RhoGAP remain unchanged. This phosphorylated RhoGAP co-immunoprecipitated with RasGAP (**Figure 1A**). In addition, we found that expression of RasGAP was not detectable in the DKO4 cell line (**Figure 1A**). It has been reported that RasGAP phosphorylation frequently occurs downstream of receptor tyrosine kinase signaling [18], [36], [37], [38], [39]. However, we found that the major tyrosine phosphorylated band that appears after immunoprecipitation of RasGAP was actually at ∼190 kDa, most likely representing RhoGAP (**Figure 1A**), indicating that RasGAP itself is not highly phosphorylated in these cells.

### RasGAP expression is lost in DKO4 cells

We found that while the DKO4 cell line expressed little to no RasGAP protein compared to DLD1, the mRNA levels of the *RASA1* gene remained at 50% of the parent cell line (**Figure 1** **, B & C**). We performed SNP arrays to examine potential copy number alterations between these isogenic cell pairs. We found very little difference between the two cell lines (data not shown). A similar result was recently found in a series of alternate clones (DKO3 and DKO1) derived from the DLD1 model [40]. Importantly, no copy number differences were seen in chromosome 5q13.3, showing that the decrease in mRNA level in DKO4 is not due to chromosomal loss.

### RasGAP mutation in CRC

**Figure 2:**
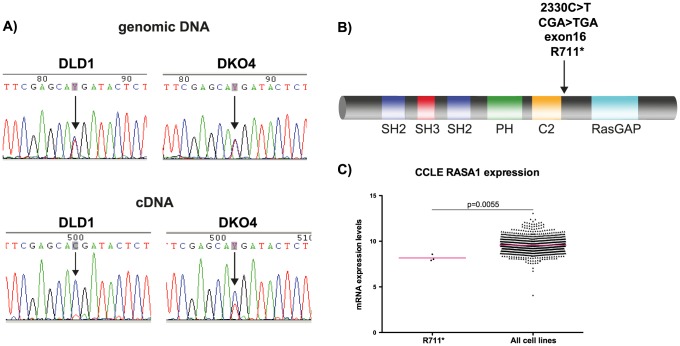
Identification of a novel truncating mutation in RASA1. A) Chromatogram showing relative intensities of each base pair after Sanger sequencing in both the genomic and cDNA derived from the cell lines. B) Illustration of location of the mutation at the RasGAP protein level. C) RASA1 expression data derived from all cell lines in the Broad Institute Cancer Cell Line Encyclopedia. Bars represent mean.

We then looked for mutations that could explain the differences in RasGAP expression level. We sequenced both the genomic DNA and the cDNA from DLD1 and DKO4 cells. We found a heterozygous point mutation in the genomic DNA of both cell lines. This C>T transition is a nonsense mutation, encoding a R709* change (**Figure 2** **, A & B**), located between the C2 and RasGAP domains of RasGAP. If the mutated gene was translated into a truncated protein, a ∼77 kDa band should have been detectable in Western blots, using a RasGAP antibody that recognizes the N-terminal portion of the protein. However, we did not see a band of this size in any cell conditions.

Interestingly, cDNA sequencing in the DLD1 and DKO4 cell lines showed that the DLD1 cell line had almost completely lost expression of the mutated gene, expressing only the wild-type, while the DKO4 cell line maintained 50% of each of the wild-type and mutant *RASA1* gene product (**Figure 2A**).

We looked for the presence of the C2330T mutation in primary tumor and metastases tissues from CRC patients, but were unable to identify this mutation in any of our samples. However, we were able to find this mutation in three cell lines from the Broad Institute Cancer Cell Line Encyclopedia (http://www.broadinstitute.org/ccle) [41]: the colorectal cancer cell lines HRT18 and HCT15, as well as the urinary tract cancer cell line 639 V. This implies that although the mutation is rare, it is present in certain types of cancer. This mutation is also not found in the human SNP database, implying that it is not found in the population at large.

To further investigate our hypothesis that the mutated *RASA1* transcript is unstable and possibly degraded, we compared the mRNA expression levels of *RASA1* in the three cell lines that contain the C2330T mutation to the remainder of the cancer cell lines in the Cell Line Encyclopedia. These three cell lines express significantly less *RASA1* than the majority of other cancer cell lines in the database (**Figure 2C**). Although not conclusive, this appears to indicate that this truncating mutation could result in decreased mRNA gene expression.

These findings suggest several levels of regulation of RasGAP, all possibly as a result of the loss of active KRAS in DKO4. First, the complete lack of mutant p120RasGAP mRNA in the DLD1 cell line as detected by sequencing indicates that either the mutant allele is not being transcribed into mRNA in this cell line, or that the mutant mRNA is very unstable and degraded immediately upon being transcribed. Interestingly, although the mutant mRNA was present in the DKO4 cell line, the 50% decrease in overall *RASA1* mRNA levels as detected by real time qPCR suggest that this mutant mRNA is also degraded, but possibly not as quickly or as efficiently as in DLD1.

In all, it appears that the loss of active KRAS in DKO4 decreases the pressure on the cell to stabilize RasGAP expression at both the mRNA and protein level; however, the mechanism by which the protein levels of RasGAP are regulated in these cell lines in still unknown.

### Regulation of RasGAP mRNA expression by mutant KRAS

**Figure 3:**
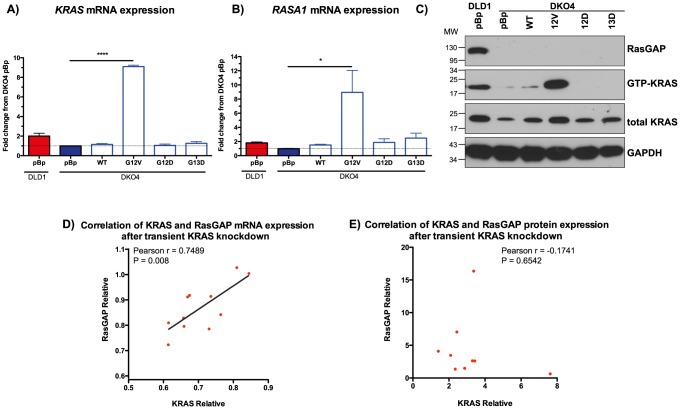
RasGAP expression is mediated in part by KRAS. Wild-type (WT) or mutant KRAS was overexpressed in DKO4 cells. mRNA was extracted from cells and quantified using rt-qPCR to measure KRAS (A) or RASA1 (B). C) Western blotting showing levels of these proteins, along with activation status of KRAS. Correlation of mRNA (D) and protein expression using densitometry analysis of Western blotting (E) of KRAS and RASA1 after knockdown of KRAS using 11 different shRNAs. For protein correlation, outliers over 3 standard deviations from the mean were excluded. All quantification is relative to empty vector. Statistical analysis of expression using unpaired t-test, ***p<0.001, *p<0.05.

To clarify the role that loss of mutant *KRAS* in DKO4 plays in the expression of RasGAP, we stably overexpressed the pBabepuro (pBp) empty vector, vector containing full length wild-type *KRAS*, or vector containing *KRAS* with point mutations in codon 12 (G12V or G12D) or codon 13 (G13D), in DKO4 cells. We hypothesized that the G13D mutation would have the largest effect in stabilizing and rescuing RasGAP expression in DKO4, due to this mutation being the one that originally occurred in this cell line. However, after repeated attempts, we were only able to overexpress the *KRAS* ^G12V^ mutant gene in this cell line (**Figure 3A**). Overexpression of G12V was accompanied by an overall increase in GTP-KRAS, as expected (**Figure 3C**). Interestingly, transient transfection of these constructs showed that KRAS was able to be overexpressed up to 7 days post transfection with all mutants, indicating that long-term expression of KRAS constructs other than G12V were not sustained in DKO4. Overexpression of *KRAS* ^G12V^ caused a significant increase in *RASA1* mRNA; however, this increase was not seen at the protein level (**Figure 3** **, B & C**). Interestingly, although we could not detect any *KRAS* overexpression with the G13D mutant, we still noted a slight increase in *RASA1* mRNA expression, although was not significant (p-value  = 0.074).

To further probe the role of active KRAS in RasGAP expression, we transiently transfected DLD1 cells with 11 separate shRNAs against *KRAS*. We found a significant correlation between the amount of *KRAS* knockdown and the decrease in *RASA1* mRNA expression compared to the non-specific shRNA control (**Figure 3D**). However, these changes were not observed at the protein level (**Figure 3E**). To ensure that KRAS shRNA knockdown did cause any significant changes in non-specific genes, **Figure S1A** shows levels of two housekeeping genes that are not affected by the knockdown. **Figure S1B** shows the Western blots from which Figure 3E was derived.

Together, these results suggest that loss of active KRAS played a partial role in the stability and/or expression of *RASA1* mRNA, although additional mechanisms are present that regulate the protein expression in this cell line.

### RasGAP overexpression rescues RasGAP activity

**Figure 4:**
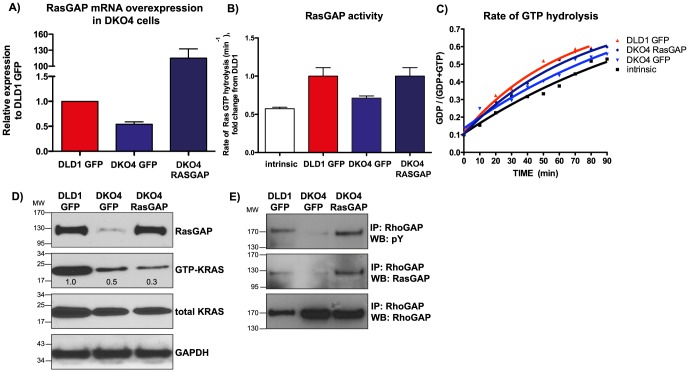
Overexpression of RasGAP expression in DLD1 cells rescues RhoGAP phosphorylation and overall GAP activity. A) mRNA expression of RasGAP after overexpression in DKO4 cells compared to the GFP vector control. B) Real-time NMR analysis of RasGAP activity, showing mean rate of GTP hydrolysis (B) and GAP activity over time (C). Each curve in (C) is derived from a single representative experiment. Error bars in (B) denote standard error of the mean (SEM). D) RAS activity assay showing levels of active KRAS after RasGAP overexpression. Numbers denote densitometry values from this blot, which is representative of three biological replicates. E) RhoGAP was immunoprecipitated from cell lines, subjected to Western blotting, then probed for total phosphotyrosine, RasGAP and RhoGAP.

To determine if any of the phenotypes attributed to loss of active KRAS in the DLD1 isogenic cell lines could be explained by the loss of RasGAP protein expression, we overexpressed RasGAP in the DKO4 cell line (**Figure 4A**). Despite our ability to get >100 fold mRNA overexpression of RasGAP in the DKO4 cell line, the resulting protein levels were similar to endogenous expression in DLD1 cells (**Figure 4D**).

To determine if overexpression of RasGAP had any effect on KRAS activity, a RAS activity assay was performed, using Raf-RBD-linked agarose beads to immunoprecipitate active KRAS (**Figure 4D**). As has been shown previously [40], KRAS overall was less active in the DKO4 cells compared to the DLD1 cells. We saw a slight but significant decrease in KRAS activity in DKO4 cells after *RasGAP* overexpression, indicating that RasGAP is able to regulate the wild-type KRAS in DKO4. To further clarify the role of RasGAP in these cells, we used a real-time NMR-based assay to determine RasGAP activity. We found that the levels of RasGAP activity were concordant with RasGAP protein expression (**Figure 4** **, B & C**). Extracts of DLD1 cells accelerated RAS GTP hydrolysis ∼1.8 fold, whereas DKO4 extracts, matched for total protein content elicited a modest 1.2 fold hydrolysis rate increase. These results were consistent with the presence of basal activity from other RasGAPs. RasGAP overexpression in DKO4 raised this rate back to ∼1.8 fold. This indicated that the ectopically expressed RasGAP is functional, as well as suggesting that RasGAP may be an important mediator of overall GAP activity in the DLD1 cell line

### RasGAP overexpression rescues RhoGAP phosphorylation and Rho-mediated phenotypes

As we showed earlier, RhoGAP phosphorylation was lost in DKO4 cells. Here we see that rescue of RasGAP expression in DKO4 cells was able to restore RhoGAP phosphorylation in these cells, as well as binding of phosphorylated RhoGAP to RasGAP (**Figure 4E**).

**Figure 5:**
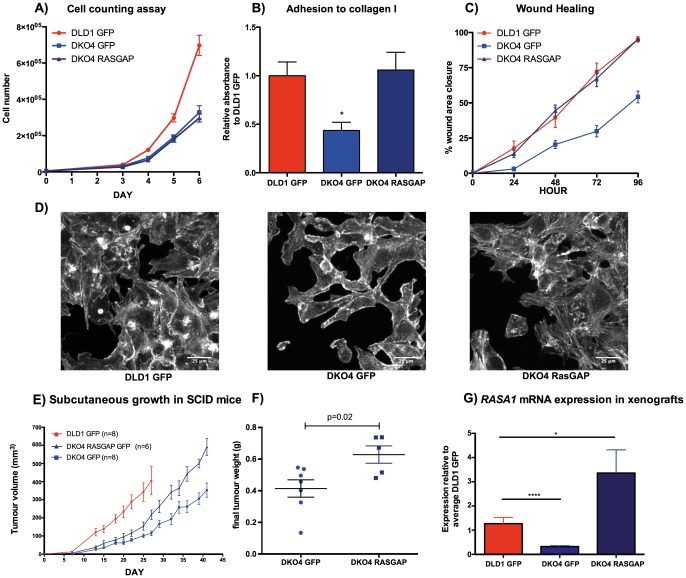
RasGAP overexpression modulates cell adhesion, cell motility, stress fiber formation and tumorigenicity. A) Cell counting assay in RasGAP overexpressing and knockdown cells. B) Cell adhesion to collagen. Statistical significance was determined by t-test: *p<0.05, ***p<0.001 C) Wound healing assay. D) Rhodamine-phalloidin staining of actin filaments after overnight adhesion to collagen. E) Tumor volume and (F) final excised tumor weight of xenograft tumors in SCID mice derived from subcutaneous injection of DLD1 empty vector (GFP), DKO4 GFP, or DKO4 overexpressing RasGAP. Number of mice used is indicated on graph. p-value calculated as indicated in materials and methods section. G) rt-qPCR analysis of RASA1 gene expression derived from xenograft tumors after excision.

RhoGAP is a key regulator of the Rho pathway, affecting phenotypes such as cell proliferation, cell adhesion to the extracellular matrix, and cell motility. These same phenotypes are differentially demonstrated in the DLD1 cell line compared to its isogenic derivatives [29]. Therefore, we were interested to know if the rescue of RhoGAP phosphorylation could also rescue these phenotypes in DKO4 cells. We found that modulating RasGAP expression did not change cell proliferation (**Figure 5A**). However, RasGAP overexpression did rescue DKO4 cell adhesion to a collagen substrate, motility, and stress fiber formation (**Figure 5** **B–D**). Together, these results indicate that rescue of RasGAP expression in DLD1 can also rescue phenotypes generally associated with Rho pathway activation.

### RasGAP and mutant KRAS together are required for full tumorigenicity of DLD1 cells

To determine if the phenotypes rescued by RasGAP overexpression in DKO4 cells could be recapitulated *in vivo*, we injected 1 million cells subcutaneously into SCID mice and measured tumor growth. Although overexpression of RasGAP did increase tumor growth significantly compared to DKO4 cells alone (**Figure 5** **, E & F**), it was not able to fully attain the growth rate of the DLD1 parent cell line, indicating that RasGAP alone is not sufficient to rescue tumorigenicity of cells that have lost active *KRAS*. The mRNA extracted from the xenografts showed that RasGAP expression remained consistent with the cells as they were prior to injection (**Figure 5G**).

## Discussion

**Figure 6:**
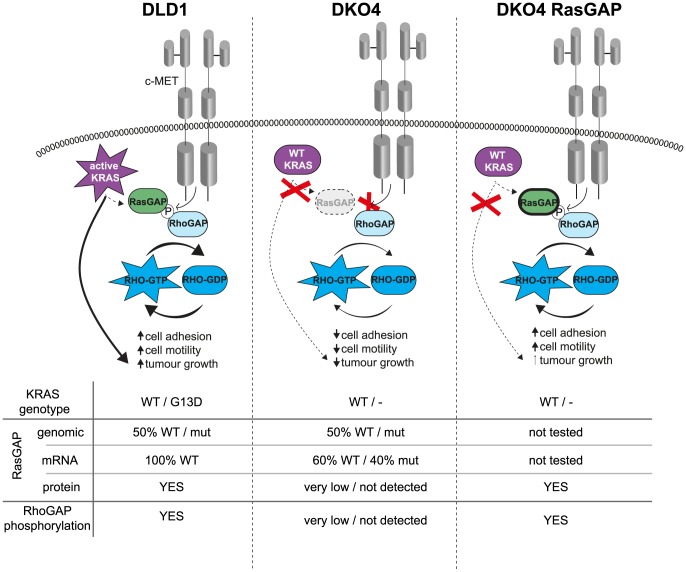
Summary of findings and proposed mechanism. In DLD1 cells, active KRAS stabilizes RasGAP expression, which in turn binds to and stabilizes RhoGAP phosphorylation. This complex then activates Rho pathway activation, either by sequestration of RhoGAP away from Rho, or by increasing Rho turnover. In DKO4 cells, RasGAP is not expressed, due in part to a truncating mutation and in part to lack of expression downstream of active KRAS. In this situation, RhoGAP is not phosphorylated, and so Rho pathway phenotypes are inactive. When RasGAP is overexpressed in DKO4, RhoGAP is once again phosphorylated and Rho pathway is active- however, lack of stabilization and/or contributing signaling pathways from active RAS means that tumorigenicity does not attain the same level as DLD1. The bottom of the figure summarizes the main characteristics of RhoGAP and RasGAP in these cell lines.

In this study, we described a role for RasGAP as an important mediator of Rho signaling and tumorigenicity in a colorectal cancer cell line, and identified mutant *KRAS* as a key contributor to this pathway (**Figure 6**). While RasGAP can act as a suppressor of RAS function by enhancing GTP hydrolysis, [6] a GAP-independent effector function has also been proposed, by virtue of its multiple binding partners [42], [43], [44], [45].

Although we were able to transiently overexpress both wild-type and various activated *KRAS* mutants in DKO4 cells, only the G12V mutation could be stably expressed. It was previously reported that Keller *et al.* failed to overexpress KRAS G12V in DKS-8, another clone of DLD1 with knockout of the KRAS mutant allele, due in part to proteasomal degradation of the mutant [46]. However, HRAS or NRAS bearing this mutation could be expressed in this cell line, suggesting that the removal of a powerful oncogene may have a myriad of effects of a cell line, causing irreversible changes that cannot be rescued by simple re-introduction [46], [47], [48]. In our hands, attempts to stably express wild-type or any codon 12 or 13 KRAS mutant (other than *KRAS* ^G12V^) failed in DKO4 cells, while transient transduction of these constructs resulted in high expression, which is consistent with previous results using DKO1 cells (another clone of DKO4) [49]. Recent work has shown that different *KRAS* mutants are associated with significantly different clinical outcomes, and yet the biological basis of these differences is just beginning to be explored [50], [51], [52]. For instance, *KRAS* ^G12V^ confers greater resistance to EGFR inhibitors in colorectal cancer, while *KRAS* ^G13D^ is associated with worse overall survival of colorectal cancer patients treated with standard chemotherapy [53].

In addition, biochemical differences may contribute to the differential expression of *KRAS* mutations in the DKO4 cell line. Using NMR to probe the GTPase activity of oncogenic RAS proteins, Smith et al. [52] showed that the G12V mutant exhibited similar intrinsic nucleotide exchange to WT, but was completely resistant to GAP-mediated GTP hydrolysis. On the other hand, intrinsic exchange of the G13D mutant was 15-fold faster than WT - this mutation retained some sensitivity to GAP-mediated hydrolysis. These results are the first steps to understanding both the pathways underlying the biology of the different RAS mutations, and the clinical differences between them.

The question of how KRAS can modulate expression of *RASA1* is an important one (**Figure 4**). Tools transcription factor prediction tool PSCAN [54] suggested that the transcription factor SPI1 may activate *RASA1* gene transcription (p = 0.02). SPI1 transcription is activated downstream of RAS-dependent AKT activation [55], and may be one mechanism by which KRAS can stabilize *RASA1* expression.

The phosphorylated tyrosines on RhoGAP responsible for RasGAP binding have not been definitively identified. One report found that phosphorylation of both tyrosines on RhoGAP, Y1087 and Y1105 [56], are responsible for binding to the tandem SH2 domains of RasGAP, while another suggested that just one site (Y1105) is sufficient [57]. However, it is generally agreed that Y1105 is the major site of tyrosine phosphorylation on RhoGAP, and the major determinant of RasGAP binding [57]. Our phospho-proteomics screen showed that Y1105 responded more strongly than Y1087 to HGF stimulation in the DLD1 cell line, although both were basally phosphorylated after serum starvation [28]. Western blots for total phosphotyrosine in RhoGAP immunoprecipitated from DLD1 detected a strong band during normal growth conditions, which did not change appreciably after HGF stimulation. Similar results were seen in mouse embryonic fibroblasts (MEF) derived from *RASA1* knockout mice, in which no phosphorylation of RhoGAP was observed *in vitro*, even after stimulation with PDGF [58]. Constitutive phosphorylation of other tyrosines may obscure changes in pY1105 by Western blot, which uses an antibody against total phospho-tyrosine. We are also not able to detect any increase in RhoGAP phosphorylation in DKO4 after HGF stimulation, suggesting that in this cell line, RasGAP is required for RhoGAP phosphorylation, both basally and in response to growth factor stimulation.

Phosphorylation of RhoGAP, and its binding to RasGAP has been shown to have conflicting roles in Rho signaling and cell migration, which likely reflect the localization of these proteins to different areas of spreading or migrating cells. In newly-adhered cells, integrin engagement leads to Src-dependent phosphorylation of RhoGAP [59] and transiently inactivation of Rho to allow Rac/Cdc42-mediated membrane protrusion at the leading edge. Later stages of migration and/or cell adhesion involve the maturation of focal adhesions and the formation of stress fibers, which are regulated by the restoration of Rho-GTP at the cell periphery of stably adherent cells, or at the leading edge of migrating cells [59]. Early studies suggested that RhoGAP phosphorylation leads to its sequestration away from Rho, allowing Rho activation and cell adhesion [25], [60], which may be a mechanism by which RasGAP mediates Rho signaling in DLD1 cells. To clarify this question, we assayed Rho in these cell lines, but could not detect any differences between DLD1 and DKO4 parental lines, nor between DKO4 cells with or without overexpressed RasGAP. In addition to having high basal levels of RhoGAP phosphorylation, the DLD1 cell line also exhibits high basal Rho activity [61], which could limit the sensitivity of the assay to detect changes. It is important to note that this method assays levels of total activated RhoA in the cell, but may be insensitive to changes in the activity or localization of a single RhoGAP against the background of many cellular RhoGAPs. Nevertheless, these events produce spatiotemporally controlled bursts of GAP activity that functionally regulate discrete sub-populations of RhoA.

The correct localization of the RhoGAP/RasGAP complex has been shown to be crucial for the proper polarization of migrating cells. MEF*^RASA1-/-^* cells showed major defects in wound healing *in vitro*, which could be partially rescued by expression of a RasGAP variant lacking the GAP domain [62]. These cells did not migrate as efficiently as wild-type MEFs, but were able to move in a fully coordinated manner, indicating that the initial polarization of cell motility requires RasGAP but not RAS, and this polarization was also dependent on p190 binding to RasGAP [62]. MEFs from RhoGAP knockout cells also showed a defect in directional cell migration [63]. In this study, we see a decrease in cell migration and cell adhesion in DKO4 cells compared with DLD1, and a concomitant increase in these phenotypes when RasGAP is re-expressed. This is consistent with a previous study that further showed that siRNA knockdown of RhoA decreased in cell migration in DLD1 but not DKO4 cells [61], further demonstrating the requirement for RasGAP, as well as active KRAS, for Rho signaling in these cells.

To explain the lack of RasGAP expression in DKO4 cells, we identified a nonsense mutation in the RASA1 gene that likely results in the decay of the messenger RNA. This specific mutation has been identified in two colon cancer cell lines, as well as urinary tract cancer cells. Interestingly, all three of these cell lines contain an activating mutation in KRAS. It is not surprising that we did not find this mutation in any of our tissue samples; for a rare mutation, our sample size was likely too small. It could be that that this mutation, although rare, is an important factor in the destabilization of *RASA1* mRNA expression in tumors. However, mutation of a neighboring arginine to a stop codon (R709*) was also recently identified in a lung carcinoma sample (COSMIC mutation 738997, obtained from the Sanger Institute Catalogue Of Somatic Mutations In Cancer web site, http://www.sanger.ac.uk/cosmic)[64] and was previously identified in two families presenting with capillary venous malformation syndrome [14]. Further analysis of these truncating mutations, and their roles in cancer and other developmental diseases, will further elaborate on the role of RasGAP in cancer.

In conclusion, this study has provided new insights into the complexity of RasGAP and KRAS signaling, and reveals a novel role for RasGAP as an effector of KRAS and Rho pathway activity in colorectal cancer. Our study also identified a novel genomic aberration with potentially significant effects on signaling studies involving the commonly used colorectal cancer cell line DLD1 and its derivatives.

## Supporting Information

Figure S1 **Controls for shRNA-mediated KRAS knockdown in DLD1 cells.** A) rt-qPCR results showing no significant expression changes in two housekeeping genes after transient KRAS knockdown. Genes are those used as loading controls in Figure 3D. B) Western blot of Figure 3E, showing protein levels after KRAS knockdown.
(EPS)
